# Rectal Bleeding and Abdominal Pain Following Vaccination in a 4-Month-Old Infant

**DOI:** 10.1155/2017/9461315

**Published:** 2017-01-09

**Authors:** Jaclyn Otero, Molly R. Posa, Maria N. Kelly

**Affiliations:** Department of Pediatrics, College of Medicine, University of Florida, Gainesville, FL, USA

## Abstract

Intussusception is one of the most frequent causes of intestinal obstruction in infants. Rotavirus vaccination has been associated with intussusception in the medical literature. We report a case of a 4-month-old female with intussusception requiring hemicolectomy one week following rotavirus vaccination. We review the pathophysiology, presentation, and management of intussusception with a distinct focus on the history of rotavirus vaccination and risks of intussusception associated with timing of rotavirus vaccine administration. The discussion makes a strong case for rotavirus vaccine counseling regarding signs of intestinal obstruction and the importance of early recognition.

## 1. Case Presentation

A 4-month-old female in foster care presents to the emergency department for evaluation of acute-onset abdominal pain with bloody bowel movements. For three to four hours prior to presentation, the infant was awakening every 10−15 minutes crying with associated tightening of her abdomen and drawing her legs inward and intermittent rectal bleeding.

She was a previously healthy full-term infant. Her routine pediatric care had been unremarkable. She had received her 4-month vaccines (Rotarix, Pediarix, Hib, and Prevnar) at a health supervision visit 7 days prior to presentation to the local emergency department

On physical examination, her vital signs were as follows: temp 97.7 F; pulse 133; weight 6.575 kg (50th percentile). She appeared well nourished on general appearance but was noted to have intermittent episodes of inconsolable crying with drawing up of her legs toward her abdomen witnessed during the exam which were followed by periods of somnolence. Her abdominal exam demonstrated a soft, nontender abdomen with normoactive bowel sounds. There were no masses or hepatosplenomegaly. Her anus was well formed and demonstrated no evidence of anal fissures, hemorrhoids, abrasions, or abnormal lesions. The remainder of her physical exam was within normal limits.

**Figure 1:**
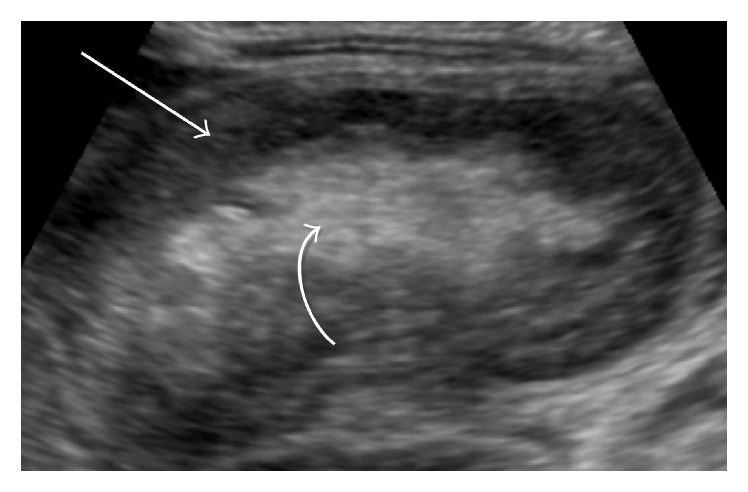
Longitudinal oblique abdominal ultrasound in the midline demonstrates an ovoid mass (curved arrow) showing the characteristic “pseudokidney” feature of intussusception.

**Figure 2:**
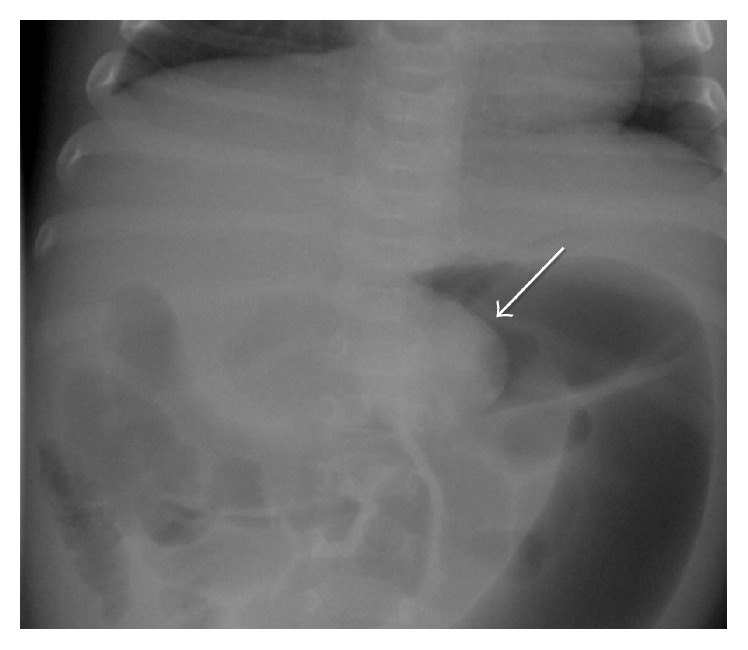
Abdominal radiograph after subsequent air enema demonstrates a “meniscus sign” of intussusception.

Due to concerns for abdominal pathology, a left lateral decubitus abdominal radiograph was obtained and demonstrated air extending throughout the gastrointestinal track with no dilated loops of bowel or free peritoneal air. The patient then underwent an abdominal ultrasound which was concerning for ileocolic intussusception (Figure 1). Following consultation with pediatric surgery, she emergently underwent an air enema reduction under fluoroscopy with air entry into the terminal ileum after several initial attempts and was hospitalized after the procedure for observation. Unfortunately, early the following morning, she presented again with intermittent fussiness and hip flexion concerning for recurrence of her intussusception. A repeat abdominal ultrasound confirmed ileocolic intussusception with a new finding of prominent ascites. An air enema under fluoroscopy was attempted again to reduce her intussusception but was unsuccessful as evidenced by the “meniscus sign” on abdominal radiograph (Figure 2). The patient was emergently taken for surgical exploration and intussusception reduction and she was found to have ischemia of the right colon and distal ileum necessitating a partial hemicolectomy and resection of the distal ileum with end-to-end ileocolonic anastomosis. The infant recovered well following the procedure and was discharged home after seven days of hospitalization with no further complications.

## 2. Discussion

Intussusception is the most common cause of intestinal obstruction in infants between 3 and 36 months of age. The incidence of intussusception is between 26 and 38 cases per 100,000 live births in the first 3 years of life [1].

Approximately 75% of intussusceptions in children are considered to be idiopathic; however, an increasing body of knowledge suggests viral triggers may play a role in some of these cases [1]. Viral infections such as enteric adenovirus can stimulate lymphatic tissue in the intestinal tract resulting in hypertrophied Peyer's patches in the terminal ileum, which may act as a lead point [2]. In approximately 25% of intussusception cases, an underlying disease process may produce a pathologic lead point [1]. Of particular note, our patient was one week after immunization for rotavirus with* Rotarix* vaccine.

Routine vaccination against rotavirus began in 1998 with the FDA approval of* RotaShield*.* RotaShield* vaccination was then voluntarily withdrawn from the market in 1999 when it was found to have an increased risk of intussusception of 1 to 2 cases per 10,000 recipients [3]. Two new rotavirus vaccinations were subsequently developed and the FDA approved* RotaTeq* and* Rotarix* in 2006 and 2008, respectively. A systematic review found that, in 31 clinical trials, there was no association with intussusception with either* RotaTeq* or* Rotarix* [4]. However, to date, positive associations have been identified in studies conducted in the United States, Singapore, Latin America, and Australia [5–10]. These studies have reported a 5- to 10-fold increase in intussusception in the first week after the first and second dose of the rotavirus vaccine. A recent analysis of the US PRISM surveillance program found both* RotaTeq* (estimated rate of 1.1–1.5 cases per 100,000) and* Rotarix* (estimated rate of 5.1 cases per 100,000 doses) were associated with intussusception less than 21 days after vaccination [3]. A study in California demonstrated a small increased risk in intussusception hospitalizations following introduction of these two new rotavirus vaccines [11]. The epidemiology of naturally occurring intussusception is known to increase significantly between the ages of 12 and 32 weeks [12]. Any potential increased risk for intussusception with rotavirus vaccines is greatest after the first dose of vaccine which is recommended to be administered at 6–15 weeks of age: the lower rates of intussusception in this age group suggests that timely administration of the vaccine would minimize the attributable risk associated with vaccination [13]. Thus, it would be interesting to look at postvaccine intussusception rates for infants who receive the rotavirus immunization at 6 weeks and 10 weeks as compared to the typical schedule of 8 weeks and 16 weeks as in theory this would decrease intussusception after vaccination. A study in Singapore, using a public health modeling analysis, illustrated the importance of ensuring that the first two doses of rotavirus vaccination are administered in infants less than 3 months old to minimize the risk of intussusception as an adverse event following rotavirus vaccination [14], which is a more stringent schedule compared to that used currently in the US where the first rotavirus vaccination must be given before 14 weeks and 6 days of age.

Patients with ileocolic intussusception classically develop a sudden onset of intermittent, severe, progressively worsening abdominal pain accompanied by inconsolable crying and drawing of the legs toward the abdomen. Vomiting may occur and become bilious. A sausage-shaped abdominal mass may be palpated in the right side of the abdomen. After intussusception occurs, the stool may contain blood and/or mucus, known as currant jelly stools.

Patients with a classic presentation of intussusception should proceed directly to hydrostatic (contrast or saline) or pneumatic enema. When the diagnosis is unclear, abdominal radiography or ultrasound can be utilized to better define abdominal pathology. However, obtaining these studies should not delay definitive treatment of intussusception. Frequently abdominal X-rays lack sensitivity in the setting of intussusception, although they can often contain nonspecific findings that may suggest or support the diagnosis. Nonspecific abdominal radiographic findings include the following: absence of bowel gas in the right lower quadrant, small bowel obstruction (dilated loops of small bowel with decompressed colon), or free intraperitoneal air in the setting of perforation. It is rare to have specific signs of intussusception on radiographs such as the* meniscus sign*, a crescent of gas within the colonic lumen outlining the apex of the intussusception, although this finding was present during our patient's intussusception recurrence (Figure 2) [15]. The classic ultrasound image of intussusception is a “coiled spring or target sign” which represents layers of the intestine within intestine. However, depending on the scanning plane, intussusception can have a variety of presentations. In the sagittal plane, the shape of the intussusceptum resembles a pseudokidney (Figure 1) [15]. Nonoperative reduction using a hydrostatic or pneumatic enema is successful in approximately 80 to 95% of patients with ileocolic intussusceptions [1]. The goal of enema therapy in reducing the intussusception is to exert pressure on the apex of the intussusceptum to force it from a pathologic position to its natural position. This is defined by successful entry of air into the terminal ileum with the disappearance of the soft tissue mass near the intussusceptum on pneumatic reduction [16]. Predictors for failing an enema reduction of intussusception include presence of symptoms over 24 hours at presentation, diarrhea, lethargy, and distal extent of intussusceptions [17]. Intussusception recurs in approximately 10% of children after successful nonoperative reduction and is most likely to occur in the first 12–24 hours, as evidenced by our patient [18]. For this reason, hospital observation for at least 24 hours is recommended even after a successful reduction. Pneumatic reduction under fluoroscopy, as used in our case, has several advantages including less radiation due to shorter fluoroscopic times [19]. A disadvantage of this technique can be seen in patients with marked amounts of gas in the small bowel proximal to intussusception prior to any procedure being performed which can make it difficult to visually confirm a successful reduction due to multiple gas filled loops in these patients [19]. Lastly, Navarro and Daneman describe previous cases where, despite air entry into the terminal ileum, there can be erroneous interpretation of a successful reduction [19]. Given the fact that our patient later presented with bowel ischemia necessitating surgery, an incomplete reduction must be considered as a causative factor. Surgical treatment is indicated for patients with suspected intussusception who are acutely ill, having evidence of perforation, incomplete reduction, or a mass lesion.

Although the risk of intussusception following rotavirus vaccination is small and does not call into question the well-documented benefits of rotavirus vaccination, parents of vaccinated infants should be informed so that they can react quickly to the first symptoms of intussusception. Early presentation would reduce the risk of failed enema reduction, prolonged hospital stays, and associated surgical complications. Future studies should be directed at investigating postvaccination intussusception rates for infants who receive rotavirus vaccination at 6 weeks and 10 weeks as compared to the typical schedule of 8 weeks and 16 weeks because in theory this would decrease intussusception rates after vaccination.
